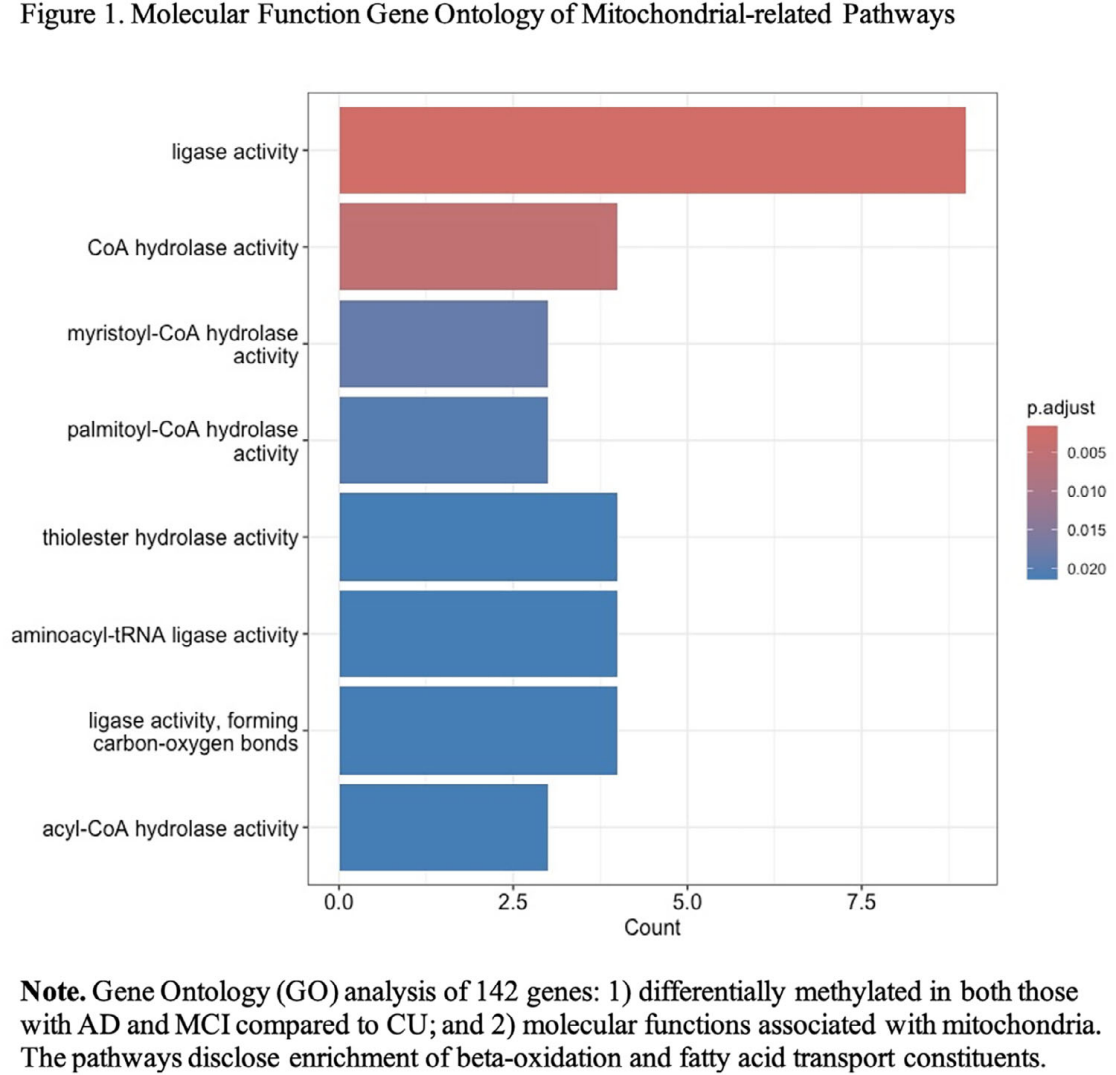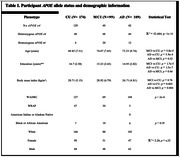# Whole genome methylation sequencing in blood reveals differential DNA methylation in pathways associated with mitochondrial function in persons with late‐onset dementia due to Alzheimer’s disease (AD)

**DOI:** 10.1002/alz.088336

**Published:** 2025-01-03

**Authors:** Alexander E Boruch, Isabelle Renteria, Andy Madrid, Coleman E Breen, Ligia A Papale, Lindsay R. Clark, Phillip E Bergmann, Sanjay Asthana, Sterling C. Johnson, Sündüz Keles, Dane B. Cook, Kirk J. Hogan, Reid S Alisch

**Affiliations:** ^1^ University of Wisconsin‐Madison, Madison, WI USA; ^2^ William S. Middleton Memorial Veterans Hospital, Madison, WI USA; ^3^ University of Wisconsin School of Medicine and Public Health, Madison, WI USA; ^4^ Geriatric Research Education and Clinical Center, William S. Middleton Memorial Veterans Hospital, Madison, WI USA; ^5^ Wisconsin Alzheimer’s Disease Research Center, University of Wisconsin‐Madison School of Medicine and Public Health, Madison, WI USA; ^6^ University of Wisconsin ‐ Madison, Madison, WI USA; ^7^ Wisconsin Alzheimer’s Disease Research Center, School of Medicine and Public Health, University of Wisconsin‐Madison, Madison, WI USA; ^8^ Geriatric Research Education and Clinical Center William S. Middleton VA Hospital, Madison, WI USA; ^9^ Wisconsin Alzheimer’s Disease Research Center, Madison, WI USA; ^10^ Wisconsin Alzheimer’s Institute, University of Wisconsin‐Madison School of Medicine and Public Health, Madison, WI USA; ^11^ School of Education, University of Wisconsin‐Madison, Madison, WI USA; ^12^ Anesthesiology, University of Wisconsin School of Medicine and Public Health, Madison, WI USA

## Abstract

**Background:**

DNA microarray‐based studies report differentially methylated positions (DMPs) in blood between cognitively unimpaired persons (CU) and persons with late‐onset dementia due to Alzheimer’s disease (AD) or Mild Cognitive Impairment (MCI) but interrogate less than 4% of the human genome. Whole genome methylation sequencing (WGMS) quantifies DNA methylation levels across the entire human genome (>25 million CpG loci). Using WGMS, we previously reported 28,038 DMPs within 2,707 genes between persons with and without AD.

**Method:**

We used WGMS to measure DNA methylation levels in nuclear genes encoding proteins that participate in mitochondrial function as cataloged in the *Human Protein Atlas* in blood samples from participants with AD (N = 109) and MCI (N = 99) compared to CU (N = 174). DNA methylation levels were compared cross‐sectionally in the Wisconsin Alzheimer’s Disease Research Center (WADRC) and the Wisconsin Registry for Alzheimer’s Prevention (WRAP) cohorts. Participant*APOE* allele and demographic information are provided in Table 1.

**Result:**

Mitochondrial‐related genes (N = 1,121) were differentially methylated (local false discovery rate [lFDR] < 0.01) in persons with AD (N = 218) and MCI (N = 421) compared to CU controls. Gene ontology pathway analyses of differentially methylated genes observed in both AD and MCI groups (N = 142) are enriched for multiple terms, including myristoyl‐CoA hydrolase, palmitoyl‐CoA hydrolase, and thiolester hydrolase activity (Figure 1). Differentially methylated genes align with differentially expressed genes that support beta‐oxidation (acyl‐CoA dehydrogenase family member 9 [*ACAD9*]) and fatty acid transport into mitochondria (carnitine palmitoyl transferase I [*CPT1A*]) in blood from persons with AD.

**Conclusion:**

Disordered pathways that participate in beta‐oxidation and fatty acid metabolism have been identified in large‐scale multi‐omic reports of AD histopathology. Present data indicate that corresponding changes in the methylome play a role in mitochondrial pathways known to contribute to the pathogenesis of AD and may serve as candidates for diagnosis and intervention.